# Sequence-to-sequence pretraining for a less-resourced Slovenian language

**DOI:** 10.3389/frai.2023.932519

**Published:** 2023-03-28

**Authors:** Matej Ulčar, Marko Robnik-Šikonja

**Affiliations:** Faculty of Computer and Information Science, University of Ljubljana, Ljubljana, Slovenia

**Keywords:** natural language processing, pretrained language models, sequence-to-sequence models, transformers, T5 model, Slovene, low-resource languages

## Abstract

**Introduction:**

Large pretrained language models have recently conquered the area of natural language processing. As an alternative to predominant masked language modeling introduced in BERT, the T5 model has introduced a more general training objective, namely sequence to sequence transformation, which more naturally fits text generation tasks. The monolingual variants of T5 models have been limited to well-resourced languages, while the massively multilingual T5 model supports 101 languages.

**Methods:**

We trained two different-sized T5-type sequence-to-sequence models for morphologically rich Slovene language with much fewer resources. We analyzed the behavior of new models on 11 tasks, eight classification ones (named entity recognition, sentiment classification, lemmatization, two question answering tasks, two natural language inference tasks, and a coreference resolution task), and three text generation tasks (text simplification and two summarization tasks on different datasets). We compared the new SloT5 models with the multilingual mT5 model, multilingual mBART-50 model, and with four encoder BERT-like models: multilingual BERT, multilingual XLM-RoBERTa, trilingual Croatian-Slovene-English BERT, and monolingual Slovene RoBERTa model.

**Results:**

Concerning the classification tasks, the SloT5 models mostly lag behind the monolingual Slovene SloBERTa model. However, these models are helpful for generative tasks and provide several useful results. In general, the size of models matters, and currently, there is not enough training data for Slovene for successful pretraining of large models.

**Discussion:**

While the results are obtained on Slovene, we believe that they may generalize to other less-resourced languages, where such models will be built. We make the training and evaluation code, as well as the trained models, publicly available.

## 1. Introduction

Recent state-of-the-art natural language processing (NLP) solutions are based on the transformer neural network architecture (Vaswani et al., 2017). The main research direction is to produce (ever larger) pretrained language models (PLMs) with billions of parameters with the objective to contain as much human knowledge as possible (Bommasani et al., 2021). Such models require large amounts of training data and are computationally expensive to train. Most very large models have been trained for English and a few high-resource languages, such as Chinese, French, or German. Massively multilingual models, trained on around 100 languages, have also been released, but their performance lags behind their monolingual and few-lingual equivalents (Ulčar et al., 2021). For some of these 100 less-resourced languages, there is a growing number of smaller models (although still in the range of a few 100 million parameters) trained on the BERT (Devlin et al., 2019) or RoBERTa (Liu et al., 2019) architecture.

BERT (Devlin et al., 2019) is a masked language model, utilizing the encoder stack of the transformer architecture to capture the semantic representation of the input text. This makes it very suitable for solving classification tasks. Other popular types of large language models are from the GPT family, such as GPT-2 (Radford et al., 2019) and GPT-3 (Brown et al., 2020), which are generative models and utilize only the decoder stack of the transformer. In contrast to these models, sequence-to-sequence (seq2seq) models such as BART (Lewis et al., 2020) and T5 (Raffel et al., 2020) utilize both encoder and decoder stacks of the transformer. Such models can treat every problem as a text-to-text transformation and solve it similarly, without adapting the training procedure for each task.

In this work, we present two new sequence-to-sequence models for the less-resourced Slovene language based on the T5 architecture and its training tasks. We aim to analyze the amount of required data for such models to be effective and the role the richer morphology plays for seq2seq models. Namely, while English has a fixed word order and relatively few word forms for each word, this is not the case for most other languages. This might not be problematic in text classification, which is a typical task for large pretrained models, while text generation tasks are more challenging for morphologically rich languages. We qualitatively and quantitatively test Slovene T5 models on three text generation tasks: lemmatization, summarization, and text simplification. We believe our results might be indicative for other less-resourced languages in terms of datasets, training, and expected results.

The work is split into further four sections. In Section 2, we summarize the related work and briefly describe the T5 architecture in Section 3, we present the architecture and training of the Slovene T5 models, which are evaluated in Section 4. We discuss the findings and their implications in Section 5.

## 2. Related work

T5 model (Raffel et al., 2020) is an encoder–decoder transformer, trained on several supervised and self-supervised pretraining tasks. The supervised tasks used were the tasks from the GLUE (Wang et al., 2018) and SuperGLUE (Wang et al., 2019) benchmarks, as well as translation and summarization tasks. The self-supervised task used was the span-corruption task. In this task, randomly selected token spans are replaced with a special mask token (a sentinel token). The goal of the task is to generate the masked spans. During pretraining, 15% of tokens were masked in spans with an average length of three tokens. The encoder stack receives the tokenized input text. The self-attention mechanism attends to the whole input in the encoder. The output of the encoder is then fed into the decoder stack, which generates the target text. A causal mask is used to prevent the self-attention mechanism in the decoder to attend to the “future” output. At each timestep, the model “sees” the whole input sequence and the part of the output sequence generated at previous timesteps. Several T5 models for English have been trained and released. They differ in size, ranging from 60 million to 11 billion parameters.

Xue et al. (2021) have trained multilingual T5 models (mT5) of various sizes. The mT5 models were trained on a large multilingual mC4 corpus, containing 101 languages, and a total of 6.3·10^12^ tokens. The Slovenian portion of the corpus contains 8.8 billion tokens. The mT5 models were trained simultaneously on all 101 languages on the span corruption task only.

BART (Lewis et al., 2020) is another popular encoder-decoder transformer model. The main difference between BART and T5 is in the choice of the pretraining tasks. Similar to T5 and mT5, BART was trained on the span corruption task. In addition, token deletion, sentence permutation, and document rotation tasks were used during pretraining. Liu et al. (2020) trained a multilingual BART (mBART) model on 25 languages, using the span corruption (masking 35% of the words) and sentence permutation training tasks. Tang et al. (2021) extended the existing mBART model to further 25 languages, thus covering 50 languages, including Slovene.

Several monolingual models, based on the T5 architecture, have been released for high-resource languages other than English, such as Chinese Mengzi (Zhang et al., 2021), Arabic AraT5 (Nagoudi et al., 2021), and Italian IT5 (Sarti and Nissim, 2022). While Nagoudi et al. (2021) observe the improvement of AraT5 over mT5 across all evaluation tasks, Sarti and Nissim (2022) note that, especially for summarization, IT5 lags behind the benchmark models. Sarti and Nissim (2022) also observed that scaling the model size does not uniformly correspond to improvements in performance. On average, the small IT5 improves the most over the comparable mT5 model, while larger IT5 models show much smaller or no improvements over comparable mT5 models and, in some cases, perform even worse than the small IT5 model.

The presented Slovene SloT5 models partially confirm and partially contradict the above findings. On one hand, we use much more challenging text classification tasks (Slovene translation of the SuperGLUE benchmark suite); therefore, the classification performance of SloT5 models consistently lags behind the BERT-like monolingual Slovene models. On the other hand, while the small SloT5 model is successful for text generation tasks, the amount of training data and training time might not be sufficient to make the large SloT5 model really competitive in the text generation tasks.

## 3. Slovene T5 models

In this section, we present the newly created Slovene T5 models (named SloT5). First, we describe the training data, followed by the description of architecture and training.

### 3.1. Training data

We trained Slovene SloT5 models on large Slovene corpora, covering a wide spectrum of genres, from fiction books to newspapers, academic language, and internet slang. We included Gigafida, Janes, KAS, SiParl, and SlWaC corpora. The corpora details are given below and summarized in Table 1.

**Table 1:** Corpora used in training of SloT5 models with their sizes in billion of tokens and words.

**Corpus**	**Genre**	**Tokens**	**Words**
Gigafida 2.0 (Krek et al., 2019b)	General language	1.33	1.11
KAS (Žagar et al., 2022)	Academic	1.70	1.33
siParl 2.0 (Pančur et al., 2020)	Parliamentary	0.24	0.20
slWaC 2.1 (Ljubešić and Erjavec, 2011)	Web crawl	0.90	0.75
Janes (Fišer et al., 2016)	Social media	0.10	0.08
- Janes-Wiki (Ljubešić et al., 2017)	– Wikipedia talk pages		
- Janes-Blog (Erjavec et al., 2017a)	– Slovene blogs		
- Janes-Forum (Erjavec et al., 2017b)	– Slovene forums		
- Janes-News (Erjavec et al., 2017c)	– Comments on online news articles		
Total		4.27	3.47
Total after deduplication		4.20	3.41

Janes subcorpora used are listed separately, but we show their combined size.

Gigafida 2.0 (Krek et al., 2020) is a general standard language corpus composed of fiction and non-fiction books, newspapers, school textbooks, and texts from the internet. The Janes corpus (Fišer et al., 2016) is a corpus of non-standard language composed of several subcorpora. Each subcorpus contains texts from a certain social medium or a group of similar media, including Twitter, blog posts, forum conversations, and user comments on news site articles. We used all Janes subcorpora, except Janes-tweet, since the contents of that subcorpus are encoded and need to be individually downloaded from Twitter, which is a lengthy process as Twitter limits the access speed. KAS (Corpus of Academic Slovene) (Erjavec et al., 2021) consists of PhD, MSc, MA, BSc, and BA theses written in Slovene between 2000 and 2018. SiParl (Pančur and Erjavec, 2020) contains minutes of Slovene national assembly between 1990 and 2018. SlWaC (Ljubešić and Erjavec, 2011) is a web corpus collected from the Slovene top-level web domain .si.

We deduplicated the corpora, using the Onion tool (Pomikálek, 2011). After the deduplication, the training dataset contained about 4 billion tokens. Finally, before training the models, we encoded the text into subword byte-pair-encodings using a sentencepiece^1^ model. We used the sentencepiece model that was trained for SloBERTa (Ulčar and Robnik-Šikonja, 2021) and contains 32,000 subword tokens in its vocabulary.

### 3.2. Architecture and training of SloT5

We trained Slovene T5 models of two different sizes: T5-sl-small and T5-sl-large. The smaller model has eight encoder and eight decoder layers, in total, about 60 million parameters. The larger model has 24 encoder and 24 decoder layers, in total, about 750 million parameters. All the models were trained in the same manner, i.e., on the same tasks with the same amount of data and the same optimizer. We compared two smaller models, which differ in the amount of training (1 or 5 epochs), and three larger models (1, 3, or 5 epochs).

We trained the models on a mixture of two self-supervised pre-training tasks: i.i.d. (independent and identically distributed) denoising and span corruption, suggested by Raffel et al. (2020). In the i.i.d. denoising task, 15% of tokens were randomly corrupted, i.e., replaced by a sentinel token. Each token has an equal probability of being corrupted (identically distributed) and all the corruption/replacing events are independent from each other. The goal of the task is to denoise the sequence by generating the correct token in place of the sentinel. This task is identical to the span corruption task, described in Section 2, except that all spans have the length of one token. The span corruption task used in training SloT5 is identical to the one used for training English T5 and multilingual mT5 models, with 15% of tokens corrupted and an average corrupted span length of 3 tokens.

The T5-sl-small_1_ and T5-sl-large_1_ models were trained for 1 million steps, with a batch size of 4,096 tokens, in total a bit less than 1 epoch. This amount of training is supposed to be sufficient, considering the ratio between the training tokens and the number of the model parameters (Komatsuzaki, 2019). In addition, we trained T5-sl-small_5_ for 763,000 steps, with a batch size of 32,768 tokens, in total around 5 epochs. T5-sl-large_3_ and T5-sl-large_5_ were trained with a batch size of 8,192 tokens, for 1.83 million steps and 3.05 million steps, respectively, which results in about 3 and 5 epochs. We trained the models on a DGX A-100 machine, using four 40 GB A100 GPUs. The training took about 3 days for T5-sl-small_1_, about 12 days for T5-sl-small_5_, about 3 weeks for T5-sl-large_1_, about 4 weeks for T5-sl-large_3_, and about 7 weeks for T5-sl-large_5_.

## 4. Evaluation

We evaluated our newly trained SloT5 models on 11 classification and generative tasks: named entity recognition, sentiment classification, lemmatization, text simplification, two summarization tasks on different datasets, and five (essentially classification) tasks from the Slovene SuperGLUE (Žagar and Robnik-Šikonja, 2022b) benchmark (two question answering, two natural language inference, and a coreference resolution task).

For classification tasks, we could use only the encoder stack of the T5 and added appropriate task-specific output heads on top of it, thus completely ignoring/bypassing the decoder stack. However, we decided to remodel the classification tasks into generative tasks, mimicking the evaluation procedure proposed by Raffel et al. (2020). Therefore, each example contained only an input string and an output string.

Next, in Section 4.1, we describe all 11 evaluation tasks and explain their preprocessing for the seq2seq models. The details of fine-tuning the SloT5 models and other compared transformer models are contained in Section 4.2. In Section 4.3, we present the results. We present qualitative analysis of the results on two tasks in Section 4.4.

### 4.1. Evaluation tasks

In this section, we describe the evaluation tasks and their preprocessing for T5 models. For the named entity recognition (NER) task and SuperGLUE tasks, we show the examples of original entries and entries preprocessed for T5 modeling in Table 2. We did not apply any special preprocessing for the sentiment analysis classification task and the generative tasks.

**Table 2:** Original examples and T5 formatted versions for each of the SuperGLUE tasks and the NER task.

BoolQ (original)	{“label”: true, “passage”: “‘Kalcijev karbid-Kalcijev karbid je kemična spojina s kemično formulo CaC. Njegova glavna uporaba v industriji je pri proizvodnji acetilena in kalcijevega cianamida. “question”: “kalcijev karbid cac2 je surovina za proizvodnjo acetilena”}
BoolQ (T5 formatted)	“Sestavek: Kalcijev karbid-Kalcijev karbid je kemična spojina s kemično formulo CaC. Njegova glavna uporaba v industriji je pri proizvodnji acetilena in kalcijevega cianamida. Vprašanje: kalcijev karbid cac2 je surovina za proizvodnjo acetilena,” “Pravilno.”
CB (original)	{“premise”: “Bil je kompleksen jezik. Ne zapisano, ampak predano. Lahko bi rekli, da je bil olupljen.”, “hypothesis”: “jezik je bil olupljen,” “label”: “entailment”}
CB (T5 formatted)	“premisa: Bil je kompleksen jezik. Ne zapisano, ampak predano. Lahko bi rekli, da je bil olupljen. hipoteza: jezik je bil olupljen,” “implikacija”
COPA (original)	{“premise”: “Moje telo je metalo senco na travo.”, “choice1”: “Sonce je vzhajalo.”, “choice2”: “Trava je bila pokošena.”, “question”: “cause,” “label”:0}
COPA (T5 formatted)	“Premisa: Moje telo je metalo senco na travo. Prva možnost: Sonce je vzhajalo. Druga možnost: Trava je bila pokošena. Kaj je vzrok?”, “prva”
RTE (original)	{“premise”: “V Iraku še ni bilo najdenega orožja za množično uničevanje.”, “hypothesis”: “V Iraku najdeno orožje za množično uničevanje.”, “label”: “not_entailment”}
RTE (T5 formatted)	“premisa: V Iraku še ni bilo najdenega orožja za množično uničevanje. hipoteza: V Iraku najdeno orožje za množično uničevanje.”, “ni implikacija”
WSC (original)	{“target”: {“span1_text”: “skodelico,” “span2_text”: “bila,” “span1_index”: 4, “span2_index”: 9}, “text”: “Iz steklenice sem v skodelico nalival vodo, dokler ni bila polna.”, “label”: true}
WSC (T5 formatted)	“WSC: Iz steklenice sem v * skodelico * nalival vodo, dokler ni # bila # polna.”, “Pravilno.”
NER	1130 4167 Bolj O
(original)	1130 4167 teoretično O
	1130 4167 pa O
	1130 4167 se O
	1130 4167 je O
	1130 4167 problema O
	1130 4167 lotil O
	1130 4167 Radical B-ORG
	1130 4167 Science I-ORG
	1130 4167 Journal I-ORG
	1130 4167 v O
	1130 4167 Londonu B-LOC
	1130 4167 . O
NER (T5 formatted)	“organizacije: Bolj teoretično pa se je problema lotil Radical Science Journal v Londonu.”, “Radical Science Journal”
	“lokacije: Bolj teoretično pa se je problema lotil Radical Science Journal v Londonu .”, “Londonu”
	“osebe: Bolj teoretično pa se je problema lotil Radical Science Journal v Londonu .”, “brez”

T5 formatted examples are in the CSV format where the first column is the input and the second the output.

#### 4.1.1. Classification tasks

Named entity recognition (NER) is a token-classification task, where each token is labeled as a named entity (NE) or not, and, if yes with the category of the NE. We used a dataset based on the ssj500k corpus v2.2 (Krek et al., 2019a). We covered three categories of NEs: persons, locations, and organizations. To our knowledge, there is no standardized way of solving the NER task using seq2seq models. We first attempted to generate labels for each token in a sentence, but the dataset was overwhelmed by label “O,” which covers all tokens that are not NEs and includes other named entity categories (e.g., products). We propose to solve the problem as a NE retrieval task. We prefixed each training sentence with each NE category, thus generating three times the number of training examples. See an example of the input and output in Table 2.

The desired output is a comma-separated list of NEs in the sentence pertaining to the prefixed category. *-If there are no NEs of the given category in a sentence, we set the output in the training set to the Slovene word “brez,” meaning “none”/“empty.” The resulting dataset still has most examples with the output “brez.” We balanced the training dataset by omitting examples without NEs with 95% probability. We followed the same procedure for the validation dataset, omitting 50% of examples without NEs. However, the test set was not modified and we kept all such examples in it.

Sentiment analysis (SA) is a sentence-level classification task composed of tweets, each labeled with one of three classes: “positive,” “negative,” or “neutral.” We used Slovenian tweets from the Twitter sentiment dataset for 15 European languages (Mozetič et al., 2016). Each class label was translated into Slovene as a single word to be generated by the model; no other formatting changes were needed.

Slovene SuperGLUE (Žagar and Robnik-Šikonja, 2022b) benchmark was translated from the English SuperGLUE benchmark (Wang et al., 2019). It contains two separate datasets: one was translated using machine translation and the other by human translators. Human translated datasets are of higher quality than machine translations but smaller in size for most tasks, as only subsets of the datasets were translated. We used five tasks from the SuperGLUE benchmark: Boolean question answering (BoolQ), Choice of Plausible Alternatives (COPA), CommitmentBank (CB), Recognizing Textual Entailment (RTE), and The Winograd Schema Challenge (WSC). For BoolQ, CB, COPA, and RTE, we used larger machine-translated datasets. For the WSC task, we used the human translated dataset (WSC is impossible to translate with machine translation tools).

BoolQ consists of triples: a passage, a question based on the passage, and an answer (true or false). In the COPA task, the goal is to pick the correct of the two given sentences, which correctly relates to the given premise and relation (cause or effect). CB and RTE datasets contain textual entailment tasks, where given a premise and a hypothesis, the goal is to predict whether the hypothesis entails the premise or not. In the WSC task, two spans in a short text are highlighted. The goal is to identify, using world knowledge and commonsense reasoning, whether both highlighted spans refer to the same entity.

SuperGLUE tasks have multiple attributes. As we can only feed a single string input to the T5 model, we have prefixed each attribute value with its key and concatenated the attributes. For example, examples in COPA task have the following attributes: *premise, choice1, choice2*, and *question*. The concatenated input string is of the format:

“*Premise: This is the example's premise. First choice: this is the value of choice1. Second choice: this is the value of choice2. What is the {cause, effect}?”*

Here, the cause and effect are the two possible values of the attribute *question*.

Examples in the WSC task contain two specifically marked texts within the input text. One span is a noun and the other a pronoun or a verb with the pronoun information implicitly included. Following the original T5 example,^2^ we indicate the first span by surrounding it with an asterisk on each side, and the second span by surrounding it with a hash symbol on each side.

#### 4.1.2. Generative tasks

We tested SloT5 models on three generative tasks: lemmatization, summarization (two datasets), and text simplification.

For lemmatization (Lem), we used a part of the Slovene ssj500k dataset (Krek et al., 2019a) included in the universal dependencies dataset. The model received an individual sentence on the input and was trained to generate the same sentence with every word lemmatized. Punctuation marks were included in the training and test sets, but we ignored them during the scoring.

For summarization, we used two news datasets: AutoSentiNews (ASN) (Bučar et al., 2018) and Slovene Press Agency (STA) news (Žagar and Robnik-Šikonja, 2022a), extracted from the Gigafida corpus. We fine-tuned and evaluated the T5 models on each dataset separately, treating each as a separate task. During fine-tuning, the input to a T5 model was an article text, and the output was its summary.

Text simplification task aims to simplify the input text to increase its readability. Common strategies include splitting long, complex sentences into multiple shorter, simple sentences, and replacing complex words with simpler, commonly used words. We utilized the Slovene text simplification dataset SloTS (Gorenc and Robnik-Šikonja, 2022), which contains sentence-aligned complex texts (original) and their simplified versions. The dataset contains entries, where a single complex sentence is repeated several times, each time paired with a different simple sentence simplifying a part of the complex sentence. We merged all such entries into a single instance, containing the complex sentence and concatenated simplified sentences. For example, three entries [(*c*_1_, *s*_1_), (*c*_1_, *s*_2_), (*c*_1_, *s*_3_)] were merged into one entry [(*c*_1_, *s*_1_*s*_2_*s*_3_)].

### 4.2. Fine-tuning T5 and compared models

We fine-tuned all compared T5 models (Slovene and multilingual) end-to-end on each task separately, using the HuggingFace transformers library.^3^ We used the AdamW optimizer with the batch size of 64. We saved the fine-tuned model after each epoch and selected the one that performed best on the validation set, using the ROUGE-L metric (Lin, 2004). We used the greedy search decoding method for the output generation and limited the maximum number of tokens in the output. We chose the maximum output length based on the target text length, shorter for the classification tasks and longer for the generative tasks. The maximum output lengths and the number of fine-tuning epochs for each task are presented in Table 3. The complete code of our experiments is publicly available.^4^

**Table 3:** Evaluation parameters and performance metrics for seq2seq and BERT models for each of the datasets.

	**Epochs**		**Output len**.		
**Task**	**seq2seq**	**BERT**	**(seq2seq)**	**Metric**	**Dataset**
BoolQ	10	100	4	Accuracy	Žagar et al., 2020
CB	15	100	6	*F*_1_	Žagar et al., 2020
COPA	15	100	6	Accuracy	Žagar et al., 2020
RTE	15	100	6	Accuracy	Žagar et al., 2020
WSC	20	100	6	Accuracy	Žagar et al., 2020
NER	20	3	64	*F*_1_	Krek et al., 2019a
SA	10	10	5	*F*_1_	Mozetič et al., 2016
Lem	15	-	512	Word/sent. acc.	Krek et al., 2019a
STA	5	-	512	ROUGE L	Krek et al., 2019b
ASN	5	-	512	ROUGE L	Bučar, 2017
SloTS	64	-	256	ROUGE L	Gorenc and Robnik-Šikonja, 2022

We compared SloT5 models with multilingual mT5 models (Xue et al., 2021), multilingual mBART-50 model (Tang et al., 2021), and with four encoder BERT-like models (described later). We fine-tuned the mT5 and mBART-50 models in the exact same manner as the SloT5 models on all 10 tasks. BERT-like models were fine-tuned on seven classification tasks, but not on the generative tasks (Lem, ASN, STA, and SloTS), as they cannot generate text. Žagar and Robnik-Šikonja (2022b) evaluated BERT-like models on the Slovene SuperGLUE benchmark. The BERT models were fine-tuned on each task individually for 100 epochs using the Jiant tool (Phang et al., 2020) with the initial learning rate of 10^−5^. Ulčar and Robnik-Šikonja (2021) evaluated BERT models supporting Slovene on NER and SA tasks. They added a softmax classification layer on top of the BERT models and fine-tuned them for 3 epochs on the NER task with a batch size of 8, and for 10 epochs on the SA task with a batch size of 32.

### 4.3. Quantitative results

We compared the results of our three monolingual SloT5 models (described in Section 3) on 11 tasks (described in Section 4.1) with two multilingual T5 models of comparable sizes, mT5-small and mT5-large (Xue et al., 2021), and with a multilingual BART model (mBART-50-large) (Tang et al., 2021). Due to their larger vocabulary sizes, mT5 models have many more parameters than comparable SloT5 models (300M vs. 60M for small and 1.2B vs. 750M for large). However, the transformer layers are identical in their number and size for both small models and for both large models. mBART-50-large model has 611M parameters, 12 encoder and 12 decoder layers, thus it lies somewhere between small and large T5 models concerning size.

For the classification tasks, we also compared the results with four encoder BERT-like models: multilingual BERT model (mBERT), multilingual XLM-RoBERTa model (XLM-R), trilingual CroSloEngual model (CSE, Croatian-Slovene-English) (Ulčar and Robnik-Šikonja, 2020), and monolingual Slovene RoBERTa model (SloBERTa).

#### 4.3.1. Classification tasks

The evaluation results on classification tasks are presented in Table 4. Some T5 models score 0 on certain SuperGLUE tasks. The reason is that the tasks were reformatted as generative tasks, and we check whether the generated text is equal to any of the class labels (in case of 0, it was not). We perform only minor post-process filtering of the generated texts, such as removing <extra_id_0> tokens added by the T5 models.

**Table 4:** Results of the compared T5 and BERT models on classification tasks.

**Model**	**BoolQ**	**CB**	**COPA**	**RTE**	**WSC**	**NER**	**SA**
Maj. class.	63.3	21.7	50.0	58.6	65.8	0.0	20.3
T5-sl-small_1_	66.6	0.0	47.6	58.6	47.9	48.1	57.4
T5-sl-small_5_	66.6	0.0	50.0	65.5	65.1	66.0	60.4
T5-sl-large_1_	70.0	48.8	51.6	58.6	61.6	53.2	59.2
T5-sl-large_3_	60.0	50.9	48.8	62.1	64.3	70.8	61.0
T5-sl-large_5_	63.3	62.2	50.6	65.5	59.6	66.9	61.8
mT5-small	70.0	0.0	0.0	55.2	0.0	2.7	56.6
mT5-large	70.0	0.0	0.0	58.6	45.2	79.0	61.9
mBART-50-large	56.7	48.6	50.0	62.1	65.1	74.7	54.7
mBERT	63.3	65.1	54.4	57.9	61.6	88.5	57.6
XLM-R	63.3	62.0	51.4	42.8	65.8	91.2	60.4
CSE BERT	63.3	59.8	55.0	53.8	56.2	92.8	61.0
SloBERTa	63.3	**68.6**	**58.2**	49.6	**73.3**	**93.3**	**62.3**

The metric for each task is shown in Table 3. The results of the best performing model for each task are in bold, and the results of the best performing seq2seq model are underlined.

On SuperGLUE tasks, all seq2seq models perform poorly. While they do outperform BERT-like models on BoolQ and RTE tasks, they barely beat the majority classifier on both tasks. The exception is the mBART-50-large model, which lags behind the majority classifier on BoolQ, and mT5-small, which performs worse than majority classifier on RTE. On RTE, T5-sl-large_5_ and T5-sl-small_5_ perform the best of all evaluated models on this task.

On NER, the multilingual mT5-small model performs poorly, while mT5-large is the best T5 model. All the T5 models lag behind the BERT-like models on the NER task. The dataset used for the SA task has a low inter-annotator agreement, limiting the overall performance. The best performing T5 models on the SA task, mT5-large and T5-sl-large_5_, perform on par and are only slightly worse than the best model on this task, SloBERTa. The small Slovene T5 models perform on par with multilingual BERT models on the SA task.

#### 4.3.2. Generative tasks

The results on the generative tasks (lemmatization, two summarization tasks, and text simplification) are shown in Table 5.

**Table 5:** Results of the compared seq2seq models on generative tasks: lemmatization (Lem), two summarization tasks (STA and ASN), and text simplification (TS).

**Model**	**Lem**	**STA**	**ASN**	**TS**
T5-sl-small_1_	90.3/37.1	20.6	20.0	29.1
T5-sl-small_5_	95.6/62.2	22.1	22.3	33.2
T5-sl-large_1_	95.5/58.5	21.5	21.9	28.3
T5-sl-large_3_	97.2/72.2	25.0	22.7	30.4
T5-sl-large_5_	97.0/73.6	25.3	22.5	30.2
mT5-small	90.0/25.5	17.9	19.2	26.3
mT5-large	**98.3/75.6**	24.5	23.2	**35.3**
mBART-50-large	97.8/73.2	**27.6**	**24.1**	33.7

The metric for each task is shown in Table 3. The results of the best performing model for each task are in bold.

While mBART-50-large does not perform very well on the classification tasks, it is the best performing model on two out of four generative tasks and the second best model on the other two tasks. If we compare only T5 models, large models consistently outperform small models, when trained on the same amount of data. The difference in performance is especially notable for the mT5 models, while it is not as big for the SloT5 models. In general, the difference in performance between T5 models is the same as observed on NER and SA tasks: mT5-large performs the best (excluding mBART), followed by T5-sl-large and T5-sl-small, while mT5-small performs the worst.

The difference in training time has a large impact for the T5-sl-small model, as with more training, the performance improves significantly on most tasks, especially the generative tasks. T5-sl-small_5_ outperforms T5-sl-large_1_ on most tasks, the exceptions are BoolQ, CB, and COPA. While longer training does improve the performance of T5-sl-large model, the difference is modest and most noticeable between one and three epochs. Surprisingly, on the tested datasets, more training of large models does not always help and it is unclear whether T5-sl-large_3_ or T5-sl-large_5_ is the best performing SloT5 model; the results depend on the task.

### 4.4. Qualitative analysis

In general, quantitative results are less informative for generative tasks compared to classification tasks. The main reason is that the evaluation metrics such as ROUGE-L score are not strongly correlated with human judgments. Below, we provide qualitative analysis of the text simplification and summarization results, while for lemmatization, we did not notice any significant patterns.

#### 4.4.1. Text simplification

We qualitatively analyzed the four best performing models on the text simplification task, according to the ROUGE-L metric. We show selected examples from the test set of SloTS dataset in Table 6. We selected examples where at least some of the models generate a reasonable simplification, and where noticeable and interesting difference between the models can be observed. The examples along with the models' outputs were translated into English, trying to mimic the original as best as possible, including the mistakes (where present). The original Slovene examples are shown in Table 7.

**Table 6:** Examples of generated simplified sentences on the text simplification task for the best performing models, translated into English.

When they noticed Matic, who was ringing the bells with all his might, they suddenly stopped and looked at him wide-eyed then asked him: “Matic, where is the fire?”	
Golden	They saw that Enaka Palica was ringing. People asked: where is the fire?
mT5-large	When people were watching Matic, they got very angry. They were asking him: Matic, where is the fire?
mBART-50-large	The judges decided, where it burns.
T5-sl-small_5_	They asked Matic: Where's the fire?
T5-sl-large_3_	When they noticed Matic, they looked at him wide-eyed.
With the access ramp, the miniature train is accessible also for people using wheelchairs.	
Golden	People with wheelchairs can also ride with Urban.
mT5-large	The miniature train is also accessible to people using wheelchairs.
mBART-50-large	People using wheelchairs can get also an access ramp.
T5-sl-small_5_	The miniature train is accessible also to disabled people.
T5-sl-large_3_	The miniature train is accessible also to people, who have a wheelchair.
And because the branches were swinging, so was the Moon above them, which was hanging from the sky. It was really bloody, and no longer fully round, and it was hanging low, like a lonely paper lantern, beaten, yet still burning above an empty fairground.	
Golden	The Moon was colored red.
mT5-large	He was bloody.
mBART-50-large	The fair was over. Now, the father has returned to the stand.
T5-sl-small_5_	At the fair was also the Moon, which was very bloody.
T5-sl-large_3_	It has never haunted again.
Above the pile of the old wall, covered with blackberries, clematis, and other shrubs, only the former castle tower was standing tall, proudly braving the hundred-year storms and gales.	
Golden	An old tower also remained.
mT5-large	On tower was many cstling plants.
mBART-50-large	In the older part was also the castle tower.
T5-sl-small_5_	There was a large tower on the courtyard.
T5-sl-large_3_	There used to be only a tower in the town.
After that I was storming around Germany and taking part in battles, which were truly beautiful.	
Golden	I liked to battle.
mT5-large	Along the way I met German of soldiers.
mBART-50-large	After that I was storming around Germany and taking part in battles.
T5-sl-small_5_	I took part in battles in Germany.
T5-sl-large_3_	I strolled a lot around Germany.
The baby was at that time already two years old and his mother was there with him on the field above the road, where she was hoeing.	
Golden	At that time the boy was 2 years old. Terba was digging on the field. The boy was along with her.
mT5-large	Mother was with him at home. She was digging and hoeing.
mBART-50-large	Mother was then already two years old.
T5-sl-small_5_	Baby was very old.
T5-sl-large_3_	There was a large baby on the field.

Grammatical and other mistakes in the model outputs were intentionally kept.

**Table 7:** Original Slovene examples of generated simplified sentences on the text simplification task for the best performing models.

Ko so zagledali Matica, ki je zvonil na vse pretege, so najprej obstali in ga debelo pogledali, nato pa so butnili vanj: "Matic, kje gori?"	
Golden	Videli so, da zvoni Enaka Palica. Ljudje so vprašali: Kje gori?
mT5-large	Ko so ljudje gledali Matica, so se zelo razjezili. Spraševali so ga: Matic, kje gori?
mBART-50-large	Sodniki so se odločili, kje gori.
T5-sl-small_5_	Matica so vprašali: Kje gori?
T5-sl-large_3_	Ko so Matica zagledali, so ga debelo pogledali.
S klančino je vlakec dostopen tudi osebam na invalidskih vozičkih.	
Golden	Z Urbanom se lahko peljejo ljudje na vozičkih.
mT5-large	Vlakec je dostopen tudi osebam na invalidskih vozičkih.
mBART-50-large	Ljudje na invalidskih vozičkih lahko dobijo tudi klančino.
T5-sl-small_5_	Vlakec je dostopen tudi invalidom.
T5-sl-large_3_	Vlakec je dostopen tudi osebam, ki imajo invalidski voziček.
In ker so se zibale veje, se je nad njimi zibal tudi mesec, ki je visel izpod neba. Bil je res krvav in ne več popolnoma okrogel, in visel je nizko, kakor bi nad praznim sejmiščem svetil še edini, neugasli, a že obtolčeni lampijon.	
Golden	Luna je bila rdeče barve.
mT5-large	Bil je krvav.
mBART-50-large	Sejma je bilo konec. Zdaj se je oče vrnil na stojnico.
T5-sl-small_5_	Na sejmu je bil tudi mesec, ki je bil zelo krvav.
T5-sl-large_3_	Nikdar več ni strašilo.
Iznad kupa starega zidovja, poraščenega z ostrogovno, srebotovino in drugim grmovjem, je molel edino še nekdanji grajski stolp kvišku, ki je ponosno kljuboval stoletnim viharjem in nevihtam.	
Golden	Ostal je tudi star stolp.
mT5-large	Na stolp je bilo veliko grdnih rastlin.
mBART-50-large	V starem delu je bil tudi grajski stolp.
T5-sl-small_5_	Na dvorišču je bil velik stolp.
T5-sl-large_3_	V mestu je bil včasih samo stolp.
Nato pa sem vihral po Nemčiji okrog ter se udeleževal bitk, ki so bile res lepe.	
Golden	Rad sem se boril.
mT5-large	Na poti sem srečal nemške vojakov.
mBART-50-large	Nato sem vihral po Nemčiji in se udeleževal bitk.
T5-sl-small_5_	Po Nemčiji sem se udeležil bitk.
T5-sl-large_3_	Po Nemčiji sem se veliko sprehajal.
Dete je bilo takrat že dve leti staro in mati je bila z njim na njivi nad cesto, kjer je okopavala.	
golden	Takrat je bil fantek star 2 leti. Terba je kopala na njivi. Fantek je bil zraven.
mT5-large	Mati je bila z njim doma. Kopala je in okopavala.
mBART-50-large	Mati je bila takrat že dve leti staro.
T5-sl-small_5_	Dete je bilo zelo staro.
T5-sl-large_3_	Na njivi je bilo veliko dete.

The English translations are contained in Table 6.

One of the difficulties of the SloTS text simplification dataset is that many complex texts are archaic and sometimes poetic. The simplified text in the dataset tends to be written in more contemporary standard language. Such examples are very difficult for the seq2seq models to simplify and they mostly generate extractive summaries, leaving out adjectives and subordinate clauses. This can best be seen in the first and the last two examples in Table 6. Another large issue in this task is the hallucination, as all the models frequently invent information not present in the original sentence. This is most commonly the case with mBART-50-large model, which is the second best performing model, according to the ROUGE-L score. T5-sl-small_5_ is the most robust model on this task. Compared to other models, it most consistently produces coherent and truthful simplifications, though it still often invents new information. On the other hand, it most frequently generates shorter outputs, leaving out information in subordinate clauses. On the examples, where all the models fail, T5-sl-small_5_ tends to perform the worst. mT5-large achieves the best ROUGE-L score. When examining its outputs, however, it seems that it either works very well (the first and the last example in Table 6) or completely fails to produce meaningful or even grammatically correct sentences (the third and fourth examples in Table 6).

When dealing with a relatively simple example with neutral language (example 2 in Table 6), all models perform very well. However, on a more complex and longer example in the same domain (not shown to save space), none of the models produce a meaningful simplification.

#### 4.4.2. Summarization

Below we summarize the qualitative findings concerning the results of different models on the ASR summarization dataset. A few illustrative examples are shown in Table 8 and their translations into English in Table 9.

**Table 8:** Original Slovene examples of generated summaries on the ASN summarization task for the best performing models.

Kandidati bodo o izboru obveščeni najpozneje do 3. aprila. Sklad je kot glavno željo prejel 60 vlog za stanovanja v Novem mestu, 53 za Postojno, 36 zaMaribor, 43 za Kranj in 15 vlog za stanovanja na Jesenicah. Povpraševanje je bilo najbolj izrazitoza lokacijah v Postojni in Novem mestu, kjer se sklad pojavlja prvič in je bil odziv zatopričakovan. Sklad bo prejete vloge podrobno preučil, izbor kupcev pa bo potekal 27. marca v navzočnostinotarja in komisije skladno z določbami razpisa. Stanovanja, ki bodo ostala neprodana v sklopu izbora, bo sklad objavil na svoji spletnistrani www.ssrs.si 3. aprila in jih sproti prodajal po pogojih razpisa od 7. aprila dalje. Kotkupci stanovanj v nadaljnji prodaji se lahko prijavijo vsi zainteresirani, ne le kupci iz izrecnonavedenih prednostnih kategorij.	
Golden	Stanovanjski sklad RS je v roku za oddajo vlog za nakup 364 tržnih stanovanj sklada v Novem mestu, Postojni, Mariboru, Kranju in na Jesenicah, ki se je iztekel v ponedeljek, prejel 207 vlog.
mT5-large	Sklad za stanovanja v Sloveniji je objavil razpis za prodajo stanovanj v nadaljnji prodaji.
mBART-50-large	Sklad za financiranje razgradnje Jedrske elektrarne Krško (JEK) je objavil razpis za prodajo stanovanj v nadaljnji prodaji.
T5-sl-small_5_	Ljubljana - Sklad za stanovanjsko gradnjo (Sklad) je danes objavil razpis za nakup stanovanj v Ljubljani, ki ga je sklad objavil v začetku marca.
T5-sl-large_3_	Stanovanjski sklad RS je objavil razpis za prodajo stanovanj v nadaljnji prodaji stanovanj.
Ivan Zidar iz SCT, prva dama Vegrada Hilda Tovšak in prvak Primorja Dušan rnigoj še vedno lahko pristanejo v priporu. Prva dva je preiskovalna sodnica včeraj sicer spustila na prostost in zahtevala, da glede odreditve pripora odloči senat treh sodnikov Okrožnega sodišča v Ljubljani. rnigoja so izpustili, ker naj bi se skliceval na imuniteto. Tožilstvo je danes v Državni svet že poslalo zahtevo, da se osumljencu imuniteto odvzame. Zidar in Tovšakova naj bi bila preveč radodarna Kazenska ovadba Zidarju, Tovšakovi in še trem drugim očita storitev kaznivega dejanja nedovoljenega dajanja daril po I. odstavku 248. člena Kazenskega zakonika, ki pravi: “Kdor osebi, ki opravlja gospodarsko dejavnost, obljubi, ponudi ali da nedovoljeno nagrado, darilo ali kakšno drugo korist zanjo ali za koga drugega zato, da bi sebi ali komu drugemu pridobil kakšno neupravičeno ugodnost pri sklenitvi posla ali storitvi iz prvega odstavka 247. člena tega zakonika (nedovoljeno sprejemanje daril), se kaznuje z zaporom od šestih mesecev do petih let. V priporu pridržali le Tomaža žiberta Kot smo že poročali je na današnji tiskovni konferenci vodja skupine tožilcev za pregon organiziranega kriminala pri Vrhovnem tožilstvu Harij Furlan povedal, da so pridržali sedem oseb, katerim očitajo dejanja dajanja in prejemanja daril. Preiskovalna sodnica je pripor odredila le članu razpisne komisije javnega podjetja Kontrola zračnega promet Tomažu žibertu.	
Golden	Ivan Zidar in Hilda Tovšak, ki sta osumljena nedovoljenega dajanja daril, se lahko še vedno znajdeta v priporu, če tako odloči senat treh sodnikov. rnigoju morajo prej odvzeti imuniteto
mT5-large	Preiskovalna sodnica je včeraj spustila Ivana Zidarja, Hildo Tovšak in Dušana rnigoja na prostost
mBART-50-large	Tožilstvo je danes v Državni svet že poslalo zahtevo, da se osumljencu imuniteto odvzame
T5-sl-small_5_	Ivan Zidar, Hilda Tovšak in Dušan rnigoj še vedno lahko pristanejo v priporu.
T5-sl-large_3_	Ivan Zidar, Hilda Tovšak in Dušan rnigoj še vedno lahko pristanejo v priporu
Norveška policija preiskuje navedbe Andersa Behringa Breivika, da ima še “dve celici”, s katerima sodeluje, poroča BBC. Navedbe je podal med prvim zaslišanjem po bombnem napadu v Oslu, na katerem je tudi priznal napad.	
Golden	Policija njegove navedbe o sodelavcih še preiskuje
mT5-large	Norveška policija preiskuje navedbe Andersa Behringa Breivika, da ima še “dve celici”, s katerima sodeluje
mBART-50-large	Navedbe je podal med prvim zaslišanjem po bombnem napadu v Oslu
T5-sl-small_5_	Norveški policisti preiskujejo navedbe Andersa Behringa Breivika, da ima še dve celici, s katerima sodeluje
T5-sl-large_3_	V prvem zaslišanju po bombnem napadu v Oslu je Breivik priznal napad

**Table 9:** Translated examples of generated summaries on the ASN summarization task for the best performing models.

Candidates will be notified of the selection by 3 April at the latest. As the main request, the Fund received 60 applications for apartments in Novo mesto, 53 for Postojna, 36 for Maribor, 43 for Kranj and 15 applications for apartments in Jesenice. Demand was most pronounced for locations in Postojna and Novo mesto, where the Fund is appearing for the first time and the response was therefore expected. The Fund will examine the received applications in detail, and the selection of buyers will take place on 27 March in the presence of a notary and a commission, in accordance with the provisions The apartments that will remain unsold as part of the selection will be published by the Fund on its website www.ssrs.si on 3 April and sold under the conditions of the tender from 7 April onwards. All interested parties, not only those from specifically listed priority categories, can apply as buyers of apartments for resale.	
Golden	The Housing Fund of the Republic of Slovenia received 207 applications in the deadline for submitting applications for the purchase of 364 market apartments in Novo mesto, Postojna, Maribor, Kranj and Jesenice, which expired on Monday.
mT5-large	The Housing Fund in Slovenia has launched a tender for the sale of apartments for resale.
mBART-50-large	The Krško Nuclear Power Plant (JEK) Fund for the Financing of Decommissioning has published a tender for the sale of apartments for resale.
T5-sl-small_5_	Ljubljana - The Housing Fund (Fund) today published a tender for the purchase of apartments in Ljubljana, which was published by the Fund in early March.
T5-sl-large_3_	The Housing Fund of the Republic of Slovenia has launched a tender for the sale of apartments for resale of apartments.
Ivan Zidar of SCT, first lady of Vegrad Hilda Tovšak and the champion of Primorje Dušan rnigoj can still be detained. The first two were released yesterday by the investigating judge and requested that a panel of three judges of the District Court in Ljubljana decide on the ordering of detention. rnigoj was released on the grounds that he had invoked immunity. The prosecutor's office has already sent a request to the National Council today to revoke the suspect's immunity. Zidar and the Tovšak were too generous Criminal charges against Zidar, Tovšak and three others accused of committing the criminal offense of illicit giving of gifts under Article 248 (1) of the Criminal Code, which says: "Whoever promises, offers or gives to a person engaged in an economic activity an unauthorized prize, a gift or some other benefit for it or for someone else in order to obtain for himself or someone else any unjustified advantage in the conclusion of a transaction or performance referred to in the first paragraph of Article 247 of this Code (unauthorized acceptance of gifts) shall be punishable by imprisonment of six months to five years." Only Tomaž žibert was detained At today's press conference, Harij Furlan, head of the organized crime prosecution team at the Supreme Prosecutor's Office, said that seven persons accused of giving and receiving gifts had been detained. The investigating judge ordered only a member of the tender commission of the public company Air Traffic Control Tomaž žibert to be detained.	
Golden	Ivan Zidar and Hilda Tovšak, suspected of illicit gift - giving, may still be placed in custody if a panel of three judges so decides. rnigoj must first be stripped of his immunity.
mT5-large	The investigating judge yesterday released Ivan Zidar, Hilda Tovšak and Dušan rnigoj
mBART-50-large	The prosecution has already sent a request to the National Council to waive the suspect's immunity.
T5-sl-small_5_	Ivan Zidar, Hilda Tovšak and Dušan rnigoj can still be placed in custody.
T5-sl-large_3_	Ivan Zidar, Hilda Tovšak and Dušan rnigoj can still be placed in custody
Norwegian police are investigating allegations made by Anders Behring Breivik that he has “two more cells” he is cooperating with, reports the BBC. He made the allegations during the first hearing after the bombing in Oslo, when he also admitted to the attack.	
Golden	Police are still investigating his allegations of coworkers
mT5-large	Norwegian police are investigating Anders Behring Breivik's claim that he has “two more cells” with which he is cooperating
mBART-50-large	He made the allegations during the first hearing after the bombing in Oslo
T5-sl-small_5_	Norwegian policemen are investigating allegations made by Anders Behring Breivik that he has two more cells with which he is cooperating
T5-sl-large_3_	In first hearing after Oslo bombing, Breivik admitted to the attack

In our summarization tasks, the ROUGE-L scores (measuring the longest n-gram overlap between the golden and generated summaries) do no accurately represent the quality of the generated summaries. The generated summaries may be correct, concise, and easy to read, yet the scores are low, because they focused on a different aspect of the news article than the provided golden summary. Generally, the provided golden summary rounds approximate the numbers and heavily paraphrases the text. The summaries generated by seq2seq models do not round the numbers and frequently copy whole sentences from the original news article. When they do paraphrase the text, they usually do it differently than the golden summaries.

A smaller T5-sl-small_5_ model commonly generates summary only from the beginning or at most the first half of the article. It is also more prone to copying whole sentences from the input text. The biggest issue of T5-sl-small_5_ is mixing up factual indicators, e.g., increase vs. decrease and most vs. least. It also tends to invent named entities, especially locations, putting many events in Ljubljana, the capital and the largest city of Slovenia, especially when no location is indicated in the original article (see the first example in Table 9). Occasionally, the model is unable to form a coherent summary.

The mBART-50-large model has similar issues as T5-sl-small_5_, but on a smaller scale. It tends not to mix factual indicators. It is the most robust model in the sense that it most frequently produces a summary that conveys the crucial information in the article. It does so by frequently copying one or two input sentences it identifies as the most important to the output and only slightly modifies them. However, it does have issues with named entities, mostly leaving them out of the generated summaries. Thus, we often cannot tell who did what or where, just that something was done (see the second and third examples in Table 9). Similar to the simplification task, mBART-50-large has the largest tendency to hallucinate (in this case, invent wrong named entities) among the analyzed models (see the first example in Table 9). It is also the model with the largest number of grammatical mistakes. When omitting subordinate clauses, adjectives and verbs, or changing the verb, the noun declensions and/or verb conjugations should also be changed to fit the new sentence, but the model leaves them in the same form as in the input text.

T5-sl-large_3_ and mT5-large both tend to generate good summaries. While we can observe many differences between the generated summaries of the two models, we cannot point out any significant qualitative differences. The differences are mainly stylistic or due to chance. T5-sl-large_3_ paraphrases the text more often (see the third example in Table 9). mT5-large, on the other hand, has more closely adapted to the summary format of the golden summaries.

## 5. Discussion and conclusion

We presented three new T5 seq2seq models for Slovene. Our comparison of monolingual and multilingual T5 and BERT-based models, applicable to Slovene, shows that in general, for classification tasks, BERT models are preferable, while for text generation tasks, T5 models show reasonable performance. The specific findings are elaborated later. While the results are obtained on Slovene, we believe that they may generalize to other less-resourced languages, where such models will be built. We make the training and evaluation code, as well as the trained models, publicly available. The code can be found at https://github.com/MatejUlcar/SloT5-tools. The released models can be found at https://www.huggingface.co/cjvt.

Both small Slovene T5 models outperform the multilingual small T5 model. However, the large multilingual model outperforms the large Slovene T5 model. Since T5-sl-small_1_ and T5-sl-large_1_ were trained for an equal amount of steps, we assume that the larger model is under-trained. Komatsuzaki (2019) and Hoffmann et al. (2022) have recently presented evidence that the amount of training needs to scale with the size of the model. However, there is no consensus on the optimal amount of training required for a given model architecture. Komatsuzaki (2019) suggests that given a fixed number of FLOPS (floating point operations per second), the optimal ratio between the number of training tokens and the number of model parameters is around 5. Hoffmann et al. (2022), on the other hand, report that the ratio should be larger, around 20. For our T5-sl-large_1_ model, this ratio is 5.5, for T5-sl-large_3_ 20, for T5-sl-large_5_ 33, for T5-sl-small_1_ 68, and for T5-sl-small_5_ 414.

T5-sl-small_5_ and T5-sl-large_1_ were trained using roughly equal amount of computing power. Since T5-sl-small_5_ outperforms T5-sl-large_1_ on most tasks, we conclude that the optimal ratio between the number of training tokens and model parameters must be higher than 5 for Slovenian T5 models.

We observe that T5-sl-small strongly outperforms multilingual mT5-small. On the other hand, mT5-large performs better than T5-sl-large. Furthermore, while T5-sl-large_1_ is clearly worse than T5-sl-large_3_ and T5-sl-large_5_, the difference between the latter two is negligible. We hypothesize that the reason for better performance of the small SloT5 model, in comparison with the mT5, is that the small models have too few parameters to successfully encode (and decode) the information in multiple languages, so a monolingual model prevails. Our hypothesis for the worse performance of T5-sl-large, compared to mT5-large and mBART-50-large, is that there was not enough training data to successfully train a model of this size, especially since further training (for more epochs) does not seem to improve the performance. mT5-large was trained on a much larger training corpus, and even its Slovenian portion was almost two times as large as our corpus.

T5 and other seq2seq models can generate text, making them suitable for solving a wider variety of NLP tasks than encoder-only models, such as BERT. However, compared to BERT-like models, T5 models seem to be much more sensitive to unbalanced classes and smaller datasets. In addition to just classifying the input, the T5 models also have to learn how to form a coherent response. This is a simple task for a limited scope of available answers, such as most SuperGLUE classification tasks, but considerably different for the NER task, which we have formatted as the text retrieval task. Still, multilingual T5 models, especially mT5-small, have often failed in learning to generate even a sensible incorrect answer, i.e., predicting any class, even incorrect. Instead, they generate answers that are not identifiable with any class value.

Fine-tuning T5 models for more epochs on a specific task might solve the issue of generating nonsensical answers; however, we may over-fit the models. Furthermore, on models that did not have this problem, we have not observed a significant change in performance on the SuperGLUE tasks when training for more than 6–8 epochs.

Although the English T5 models (Raffel et al., 2020) were pretrained on multiple tasks, including the SuperGLUE tasks, the authors fine-tuned the pretrained models for each task during the evaluation. Their results show that the largest T5 models achieve better results than the RoBERTa_*LARGE*_ (Liu et al., 2019) baseline. However, those models are of an order of magnitude larger than the baseline model. Comparing the performance of similarly sized models, the RoBERTa model outperforms T5 on all SuperGLUE tasks. Xue et al. (2021) reported much better performance of mT5 models compared to multilingual BERT-like models in the zero-shot cross-lingual setting. In a monolingual setting, only the largest (3B and 11B) mT5 models outperform mBERT on the NER task. On the other hand, on the question answering task, all mT5 models (except for the smallest mT5-small) outperform the mBERT score. This is in line with our findings, where we observe a slight improvement of T5 models over BERT-like models on the question answering BoolQ task but worse performance on other SuperGLUE tasks.

In future work, we will try to obtain more Slovene data and retrain the large Slovene T5 model to analyze the behavior of the generative models with respect to the size of the training data. As text generation seems to be a stronger side of T5 models, we will expand the set of tackled tasks to paraphrasing and grammar correction tasks.

## Data availability statement

The original contributions presented in the study are included in the article/supplementary material, further inquiries can be directed to the corresponding author.

## Author contributions

MU has conducted the computational part of the work and written the first draft of the article. MR-Š has lead the research and contributed to writing. All authors contributed to the article and approved the submitted version.

## Footnotes

^1^ https://github.com/google/sentencepiece

^2^ https://github.com/google-research/text-to-text-transfer-transformer/blob/main/t5/data/preprocessors.py

^3^ https://huggingface.co/

^4^ https://github.com/MatejUlcar/SloT5-tools
